# Draft Genome Sequences for Oil-Degrading Bacterial Strains from Beach Sands Impacted by the Deepwater Horizon Oil Spill

**DOI:** 10.1128/genomeA.01015-13

**Published:** 2013-12-19

**Authors:** Will A. Overholt, Stefan J. Green, Kala P. Marks, Raghavee Venkatraman, Om Prakash, Joel E. Kostka

**Affiliations:** School of Biology, Georgia Institute of Technology, Atlanta, Georgia, USAa; School of Earth & Atmospheric Sciences, Georgia Institute of Technology, Atlanta, Georgia, USAb; DNA Services Facility, University of Illinois at Chicago, Chicago, Illinois, USAc; Department of Biological Sciences, University of Illinois at Chicago, Chicago, Illinois, USAd; Department of Bioengineering, University of Illinois at Chicago, Chicago, Illinois, USAe; National Centre for Cell Science, Pune, Indiaf

## Abstract

We report the draft genome sequences of 10 proteobacterial strains isolated from beach sands contaminated with crude oil discharged from the Deepwater Horizon spill, which were cultivated under aerobic and anaerobic conditions with crude oil as the sole carbon source. All strains contain multiple putative genes belonging to hydrocarbon degradation pathways.

## GENOME ANNOUNCEMENT

The Deepwater Horizon (DWH) well blowout resulted in the largest accidental release of oil into the marine environment to date (1). The ultimate fate of the majority of hydrocarbons in the marine environment is degradation by microbes, and cultivated representatives are required to interrogate the mechanisms by which these organisms catalyze hydrocarbon degradation (2, 3). In this study, 24 bacterial strains were isolated from oil-contaminated beach sediments sampled at Pensacola Beach, FL (30.32563°N, 87.17441°W), and Elmer’s Island Beach, LA (29.17845°N, 90.06923°W), on 30 July 2010 and 3 June 2010, respectively. Both of these beaches were impacted by Macondo (Mississippi Canyon Block 252 [MC252]) oil that came ashore during the Deepwater Horizon oil discharge event (4, 5). All strains were isolated in a modified minimal artificial seawater medium with MC252 crude oil as the sole carbon source under aerobic conditions (for *Acinetobacter* sp. strain COS3, *Alcanivorax* sp. strain P2S70, *Marinobacter* sp. strain C1S70, *Labrenzia* sp. strain C1B10, and *Marinobacter* sp. strain C1B70) or under anaerobic conditions with nitrate as the sole electron acceptor (for *Marinobacter* sp. strain EN3, *Marinobacter* sp. strain ES1, *Alcanivorax* sp. strain PN-3, *Marinobacter* sp. strain EVN1, and *Halomonas* sp. strain PBN3) (6). Our previous work demonstrated that several of the isolates are representative of the dominant microbial populations detected *in situ* in the same contaminated sands (4). Oil degradation capabilities were confirmed for a subset of these strains from the quantification of residual oil and concomitant growth as optical density and cellular protein (4). The initial physiological characterization revealed contrasts between strains, suggesting niche specialization in carbon and major nutrient metabolism (3).

Here, we report the draft genome sequences for 10 of these bacterial strains belonging to five genera within the *Alphaproteobacteria* and *Gammaproteobacteria* (Table 1). Among the 24 isolates, these strains were selected based on their environmental abundances, physiologies, and genome representation within the scientific community. Genomic DNA was isolated and sequenced on an Illumina HiSeq 2000 instrument, employing paired-end 100-base reads in a method similar to that of Shesmesh et al. (7). For each genome, a total of 12 to 19 million reads (6 to 9.5 million reads in pairs) of 100 bases were acquired (~1.2 to 1.9 Gb of sequence data per isolate). *De novo* assembly of paired-end reads was performed within the software package CLC Genomics Workbench version 6.0 (CLC bio, Cambridge, MA). The contigs were successfully used for annotation and gene prediction by RAST (8) and the NCBI Prokaryotic Genomes Automatic Annotation Pipeline (PGAAP), version 2.0. The abundance of genes involved in hydrocarbon degradation in each strain was determined using RAST annotation.

**TABLE 1:** Summary of the whole-genome sequence information for 10 strains isolated from oil-contaminated beach sands with MC252 crude oil as the sole carbon source

Class	Genus	Strain	No. of contigs	Total assembly (reads)	N_50_ (bp)	Approx avg coverage	Isolation source^*a*^	Isolation conditions^*b*^	GenBank accession no.	Total no. of putative genes	Total no. of putative genes for alkane degradation	Total no. of putative genes for aromatic degradation
*Gammaproteobacteria*	*Alcanivorax*	PN-3	104	4,697,326	125,004	200×	PB	N	AXBX00000000	4,338	34	17
*Gammaproteobacteria*	*Alcanivorax*	P2S70	76	3,636,972	181,484	200×	PB	A	AXBZ00000000	3,363	17	14
*Gammaproteobacteria*	*Marinobacter*	EN3	106	3,979,725	212,596	250×	EB	N	AXCC00000000	3,688	19	8
*Gammaproteobacteria*	*Marinobacter*	ES-1	117	3,559,739	86,139	300×	EB	N	AXBV00000000	3,303	16	15
*Gammaproteobacteria*	*Marinobacter*	EVN1	71	4,301,017	273,738	250×	PB	N	AXCB00000000	3,978	25	15
*Gammaproteobacteria*	*Marinobacter*	C1S70	105	4,126,405	150,583	250×	PB	A	AXBW00000000	3,830	21	8
*Gammaproteobacteria*	*Acinetobacter*	COS3	80	3,440,295	159,063	400×	EB	A	AXCD00000000	3,293	21	20
*Gammaproteobacteria*	*Halomonas*	PBN3	228	3,668,536	46,654	200×	PB	N	AXCA00000000	3,419	24	10
*Alphaproteobacteria*	*Labrenzia*	C1B10	70	6,831,407	478,574	150×	PB	A	AXBY00000000	6,539	39	14
*Alphaproteobacteria*	*Labrenzia*	C1B70	63	6,831,462	478,574	150×	PB	A	AXCE00000000	6,541	39	14

a Strains were isolated from contaminated sands from Pensacola Beach, FL (PB), or from Elmer’s Island Beach, LA (EB).

b Strains were isolated either under aerobic (A) conditions or under anaerobic (N) conditions, with nitrate as the sole electron donor.

The draft genome sequences were assembled in order to understand the metabolic potential for degrading crude oil components at the strain level, as well as to provide reference genomes in support of metagenomic work to characterize microbial populations in beach sands contaminated during the Deepwater Horizon discharge. Furthermore, we seek to compare the underlying genetic basis for hydrocarbon degradation across diverse bacterial strains competing in the same temporal and spatial environment. Substantial variation in the assembly and number of hydrocarbon degradation genes among strains of the same genus (*Alcanivorax* and *Marinobacter*; see Table 1) suggests strain-specific differences in metabolic and hydrocarbon degradation potential. Further, bacterial isolates from the genus *Labrenzia* (*Alphaproteobacteria*) represent the first genome sequences of the group for strains grown on crude oil.

### Nucleotide sequence accession numbers.

The draft genome sequences of the strains obtained in this study have been deposited in GenBank as part of BioProject no. 217943, with individual genome sequences submitted as whole-genome shotgun projects in GenBank under accession no. AXBX00000000 (*Alcanivorax* PN-3), AXBZ00000000 (*Alcanivorax* P2S70), AXCC00000000 (*Marinobacter* EN3), AXBV00000000 (*Marinobacter* ES-1), AXCB00000000 (*Marinobacter* EVN1), AXBW00000000 (*Marinobacter* C1S70), AXCD00000000 (*Acinetobacter* COS3), AXCA00000000 (*Halomonas* PBN3), AXBY00000000 (*Labrenzia* C1B10), and AXCE00000000 (*Labrenzia* C1B70).
